# Men’s Sexual Faithfulness Judgments May Contain a Kernel of Truth

**DOI:** 10.1371/journal.pone.0134007

**Published:** 2015-08-05

**Authors:** Samantha Leivers, Leigh W. Simmons, Gillian Rhodes

**Affiliations:** 1 Centre for Evolutionary Biology & School of Animal Biology, University of Western Australia, Crawley, 6009, Australia; 2 ARC Centre of Excellence in Cognition and its Disorders, School of Psychology, University of Western Australia, Crawley, 6009, Australia; Brock University, CANADA

## Abstract

Mechanisms enabling men to identify women likely to engage in extra-pair copulations (EPCs) would be advantageous in avoiding cuckoldry. Men’s judgments of female sexual faithfulness often show high consensus, but accuracy appears poor. We examined whether accuracy of these judgments made to images of women could be improved through i) employing a forced choice task, in which men were asked to select the more faithful of two women and/or ii) providing men with full person images. In Experiment 1, men rated 34 women, for whom we had self-reported EPC behavior, on faithfulness, trustworthiness or attractiveness from either face or full person photographs. They then completed a forced choice task, selecting the more faithful of two woman from 17 pairs of images, each containing one woman who had reported no EPCs and one who had reported two or more EPCs. Men were unable to rate faithfulness with any accuracy, replicating previous findings. However, when asked to choose the more faithful of two women, they performed significantly above chance, although the ability to judge faithfulness at above-chance levels did not generalize to all pairs of women. Although there was no significant difference in accuracy for face and full person image pairs, only judgments from faces were significantly above chance. In Experiment 2, we showed that this accuracy for faces was repeatable in a new sample of men. We also showed that individual variation in accuracy was unrelated to variation in preferences for faithfulness in a long-term partner. Overall, these results show that men’s judgments of faithfulness made from faces of unfamiliar women may contain a kernel of truth.

## Introduction

Across species, males and females show preferences for traits in potential partners that are believed to advertise mate quality [1]. In humans, women value traits such as dominance and the ability to accrue resources, whereas men predominantly value youth and attractiveness [2–4]. However, one trait that both sexes value in a potential mate is faithfulness (i.e. being sexual exclusive to one's partner, [5]). In socially monogamous species- such as humans- where both the male and female invest resources in offspring for an extended period of time, pairing with a mate who engages in extra-pair copulations (EPCs) may result in fitness costs for either sex. However, males are likely to suffer the greater evolutionary cost as they risk raising genetically unrelated offspring (cuckoldry).

Due to the significant fitness cost associated with cuckoldry, it would be adaptive for men to have evolved the ability to predict or detect unfaithfulness in a potential partner. There is some evidence that men can accurately judge female faithfulness [6,7], based on behaviors of, and interactions with, their partners and these judgments have been linked to the performance of anti-cuckoldry behaviors in men, including aggression, mate guarding and sexual coercion [8,9]. However, people can make many trait judgments from only limited sensory information-such as images- and some of these judgments show real life accuracy [10,11]. Faithfulness can also be judged from limited sensory information [12–14]. However, although men often show high consensus on these judgments, little is known about whether these first impressions of faithfulness are accurate.

### Accuracy of faithfulness judgments

Judgments of faithfulness can only be adaptive if they are accurate and thus prevent cuckoldry, but the accuracy of faithfulness judgments is relatively unknown. Socio-sexual orientation is often linked to attitudes regarding faithfulness as it reflects an individual’s willingness to engage in sex outside of a committed relationship. Individuals with unrestricted sociosexual orientations self-report being more willing to engage in EPCs than those with a restricted socio-sexual orientation [15,16]. A number of studies have demonstrated that people can accurately predict the socio-sexual attitudes of unfamiliar individuals from facial images alone [17,18]. However, although this research suggests that faithfulness may be predicted from images alone, there has been little research investigating the relationship between perceived faithfulness and actual EPC behavior.

One study that has directly compared perceived and actual faithfulness found that men’s ratings of faithfulness made from women’s faces did not correlate with the self-reported EPC behavior of those women [13]. Here we test whether men show accuracy in their judgments of faithfulness when asked to choose the more faithful of two women who differ in self-reported EPC behavior in a forced choice task. Within the perceptual and cognitive sciences, forced choice tasks are widely used to test for discrimination ability [19,20,21]. They can potentially reveal sensitivity to subtle stimulus differences that would not be distinguished in a rating task. Thus, we ask whether men can show accuracy in their judgments of faithfulness in a forced choice task that would otherwise go undetected in a rating task.

We also asked whether accuracy could be improved by showing full person images of the women. We often use information from the body as well as the face when making judgments of people. For example, the face and body make independent contributions to judgments of attractiveness [22,23]. Here we asked whether men can assess faithfulness more accurately from full person than from face only images.

We also asked whether individual variation in men’s preference for faithfulness in a long-term partner was related to the accuracy of their faithfulness judgments. Just as men show individual differences in the traits they prefer in potential partners [24], they also vary in their preference for faithfulness. Specifically, men who have a more restricted sociosexual orientation show a greater preference for faithfulness in a potential partner than men with unrestricted sociosexual orientations [25]. Therefore, one might expect those who place greater value on faithfulness to be more accurate in their judgments of faithfulness.

### Visual cues and traits influencing faithfulness judgments

Little is known about the visual cues that men may use to judge faithfulness. One cue that men appear to use is female attractiveness, with more attractive women often rated as less likely to be faithful [12–14]. Judging attractive women as less faithful might result in accuracy because attractive women are preferred as sexual partners and thus may have more opportunity to engage in extra-pair copulations. Indeed, attractive women report having men attempt to poach them from relationships more often than less attractive women [4,26,27]. However, data are mixed as to whether attractive individuals actually are less faithful [13,28,29].

Judgments of other traits might also influence judgments of faithfulness. For example, men could potentially use perceived trustworthiness to make judgments of faithfulness. Trustworthiness has received considerable attention in trait-judgment research [30–33]. Trustworthiness is a trait with many dimensions. For example, faithfulness could be considered an aspect of ‘sexual’ trustworthiness and, if an individual is considered generally trustworthy, they may in turn be considered to be more faithful. However, Rhodes et al. [13] found that women’s judgments of male trustworthiness were independent of their judgments of male faithfulness. Since women showed accuracy in their judgments of faithfulness in that study, the findings suggested that trustworthiness judgments do not aid in making accurate judgments of faithfulness. Men showed no accuracy in their ratings of faithfulness in Rhodes et al. [13], so the extent to which perceived trustworthiness might be related to men’s accurate judgments of faithfulness is unknown.

The extent to which men may use judgments of attractiveness and trustworthiness to make *accurate* judgments of faithfulness requires further research, and is examined in this study.

### The current study

We investigated whether men are able to make accurate judgments of faithfulness from images of women and examined the cues and traits they may use to make these judgments. In Experiment 1, we asked whether use of a forced choice task, which allows for direct comparison between pairs of women, increases accuracy above the chance levels found when rating individual face images in Rhodes et al. [13]. We included full person images as well as face images, to determine whether accuracy would improve when the body as well as the face was shown. We also examined how judgments of attractiveness and trustworthiness relate to the accuracy of faithfulness judgments. In Experiment 2, we attempted to replicate the accuracy in judgments to faces found in Experiment 1 with a new sample of participants and investigate whether individual variation in preferences for faithfulness were related to men’s accuracy in faithfulness judgments.

## Experiment 1

Participants were asked to complete two tasks: a ratings task where they rated individual women for faithfulness, and a forced choice task, in which they chose the more faithful of two women who differed in self-reported EPCs. We included the ratings task to allow direct comparison with the findings of Rhodes et al. [11]. Following Rhodes et al. [11], we did not expect to find any accuracy in this task. We included the forced choice task to determine whether men can judge faithfulness with any accuracy when they have the opportunity to directly compare women. We had self-reported EPC behavior for each of the models judged which allowed us to examine the accuracy of these judgments in both tasks [34]. Forty-three participants completed the two tasks using either face images or full person images to determine whether the use of full person images (face and body) can improve accuracy of faithfulness judgments. In order to avoid possible familiarity and carry-over effects on trait judgments [35,36], we had additional separate groups of male participants rate the female models for attractiveness and perceived trustworthiness from face or full-person images to determine whether these judgments relate to accurate judgments of faithfulness. Individuals show high consensus on trait judgments, including judgments of trustworthiness and faithfulness [31,37,38], so having judgments made for each trait by separate groups of men is a reasonable approach for this type of research.

### Materials and methods

#### Participants

Eighty-seven self-reported heterosexual, male participants aged between 18 and 35 years of age and of Western European descent were recruited from the University of Western Australia community and were awarded either psychology course credits or were remunerated with AU$5 for their participation.

#### Ethics statement

Ethics approval for this research was granted by the University of Western Australia Human Ethics Research Committee (project number RA/4/1/4681). Participants read an information sheet detailing their role in the study and provided written consent prior to commencing the study.

#### Ratings task

We obtained colored, front-view face and full person (face and body) digital photographs of 34 heterosexual women of Western European descent aged between 20 and 42 years from the database described in Rhodes et al. [25] and also used by Rhodes et al. [11]. For each of these women, we had self-reported EPC behavior. In order to maximize honest reporting, they were informed that all responses were confidential and would be stored in a locked box. They also completed the questionnaire in isolation and could only be identified via a self-selected PIN [25]. Photographs of each woman were taken from a fixed distance under symmetrical lighting conditions. Models stood with their arms relaxed by their side, with a neutral expression and no make-up. In order to standardize images, Adobe Photoshop CS3 was used to color clothing black, remove jewelry, and block out background features. Face images were scaled to a height of 420 pixels and width of 320 pixels and were surrounded by a black oval mask that covered most of the hair. Face images were viewed at an approximate distance of 50cm, at a vertical visual angle of approximately 8.3 degrees and a horizontal visual angle of approximately 6.3 degrees. Full person images were scaled to a height of 768 pixels and width of 512 pixels, and were viewed at an approximate distance of 50cm at a vertical visual angle of approximately 17.0 degrees and a horizontal visual angle of approximately 5.0 degrees. Photographs were rotated so that both pupil centers were located on the same *y*-axis and were presented at a resolution of 72 pixels/inch.

From these images, we obtained ratings of faithfulness, attractiveness and perceived trustworthiness from full person or face images. Attractiveness ratings of the face images were available from Rhodes et al. [25] so were not collected again. Participants were assigned to one of the other five tasks. The ratings task began with three practice trials using three alternative randomly selected models from the database [25]. A full person *or* face image of each model was then presented for 2 s followed by a response screen asking participants to make a rating of how faithful *or* trustworthy *or* attractive (full person only) they perceived the model to be on a Likert scale from 1 (‘Not at all’) to 7 (‘Extremely’), which was made by pressing the corresponding key on the keyboard. After making their rating, they were instructed to press the space bar to start the next trial. Participants completed this process for all 34 models and the presentation order of the models was randomized for each participant.

#### Faithfulness forced choice task

For the forced choice task, the 34 models were matched into 17 pairs. The women in each pair were of similar age (±2 years) with one woman reporting engaging in two or more EPCs (hereafter ‘unfaithful model’) and the other reporting having never engaged in an EPC (hereafter ‘faithful model’). The pair was displayed with one image presented on the left of the screen and the other on the right. Participants made their judgments from either face or full person images (same image type as the ratings task). Face images were presented approximately 1–1.5 inches apart and full person images approximately 2^3/4^–3 inches apart.

In order to become familiar with the forced choice task, prior to the experiment beginning participants were presented with three practice trials using six randomly selected models from the database that were not in the experimental stimuli pairs [25]. After these practice trials, participants completed the experimental trials. Each trial started with a fixation cross in the middle of the display screen for 500ms followed by a pair of full person or face images. The images were presented for 4 s with the unfaithful model appearing on the left of the screen for nine trials and on the right for the other eight trials. After each pair, a response screen appeared asking the participant to choose which woman was the more faithful. Participants chose the individual on the left of the screen (by pressing ‘Z’ on the keyboard, labeled as ‘Left’) or the right (by pressing ‘M’ on the keyboard, labeled as ‘Right’) and then initiated the next trial by pressing the space bar. Participants completed the trials in a random order.

#### General procedure

All testing took place on a MacBook Pro, 15 inch, 1440 x 900 pixel resolution screen. All experimental tasks were programmed and performed using SuperLab 4.

Participants first completed the ratings task, in which they were assigned to one of five alternative judgments: faithfulness from full person images (*N* = 22), faithfulness from face images (*N* = 21), trustworthiness from full person images (*N* = 14), trustworthiness from face images (*N* = 15) or attractiveness from full person images (*N* = 15). Once the ratings task was complete, participants rating faithfulness had a one minute break before moving onto the forced choice task.

The participants who rated faithfulness (*N* = 43) then completed the forced choice task in which they were presented with 17 pairs of women and were instructed to choose the more faithful of the two either from face or full person images (same image type as the ratings task). Once the forced choice task was completed, participants were thanked for their time and debriefed.

### Results and discussion

Where variables did not meet the assumption of normality according to a Kolmogorov Smirnov test, parametric analysis was still used because *z* scores calculated from skewness and kurtosis values were less than 1.96 [39]. Non-parametric analyses did not alter our results and are presented in (S1 File) to allow comparison.

#### Accuracy of faithfulness judgments in forced choice task

The proportion of correct choices was defined as the proportion of trials on which the participant correctly chose the faithful model. An independent samples *t* test showed no significant difference between the proportion of correct choices for face and full person images (*t*_41_ = 0.66, *p* = .515, effect size: *r* = 0.10, 95% CI = -0.21–0.39). Overall performance was significantly above chance (0.5) (one sample t test: *t*_42_ = 3.10, *p* = .003, *X*±SD = 0.55±0.11) and the effect size was medium-large (*r* = 0.43, 95% CI = 0.15–0.65). Although there was no significant difference in performance on faces and full person images, planned t-tests showed that performance was significantly above chance for judgments from face images (one sample t test: *t*_20_ = 2.46, *p* = .023, *X*±SD = 0.57±0.12, effect size: *r* = 0.48, 95% CI = 0.06–0.76) but did not reach significance from full person images (one sample t test: *t*_21_ = 1.87, *p* = .075, *X*±SD = 0.54±0.11, effect size: *r* = 0.38, 95% CI = -0.05–0.70). Nevertheless effect sizes were moderate in both cases.

The proportion of times that participants correctly chose the most faithful model from each of the 17 pairs was calculated and we found considerable variation between the 17 pairs of women in how likely participants were to choose the most faithful model of the pair (proportion of correct responses ranging from 0.29–0.86, *X*±SD = 0.57±0.18). Although the average proportion of correct responses fell below chance level (0.5) for only six of the 17 pairs, the mean proportion of correct responses was not significantly different from chance (one sample t test: *t*_16_ = 1.61, *p* = .127, effect size: *r* = 0.37, 95% CI = -0.15–0.73).

These results show that men’s judgments of faithfulness from faces have a kernel of truth. However, although above-chance accuracy generalized across participants, it did not generalize to all pairs of faces.

#### Accuracy of faithfulness judgments in ratings task

Ratings of each judgment (faithfulness, attractiveness or trustworthiness) made from each image type (face or full person) were made by between 12 and 22 participants. Consensus for all judgments was high (Cronbach’s *α*: face/faith = 0.81, full/faith = 0.88, face/trust = 0.87, full/trust = 0.87, face/attract = 0.81, full/attract = 0.97) so participant ratings were averaged to produce a mean rating of faithfulness, trustworthiness and attractiveness of the face and full person images for each of the 34 models. Correlations between ratings of faithfulness, trustworthiness and attractiveness made from both face and full person images are presented in Table 1 and corroborate those reported in Rhodes et al. [11].

**Table 1:** Correlations between ratings of faithfulness, trustworthiness and attractiveness made from face and full person images (Pearson’s *r* are shown above the diagonal and Spearman’s *r* shown below the diagonal for comparison) .

		Face			Full person		
		Faith	Trust	Attract	Faith	Trust	Attract
**Face**	Faith		.487**	-.424* †	.611**	.429*	-.278
	Trust	.526**		.433*	.122	.501**	.383*
	Attract	-.300	.443**		-.450**	.111	.781**
**Full person**	Faith	.605**	.167	-.417*		.474**	-.453**
	Trust	.354*	.484**	.124	.448**		.337♯
	Attract	-.190	.327	.732**	-.413*	.329	

** p< .01

* p< .05

^♯^p = .052

^†^ Once an outlier with a leverage value greater than twice the average leverage value was removed from analysis, this correlation became non-significant (*r* = -.267, *p* = 0.134).

To examine men’s accuracy in faithfulness judgments in the ratings task, we examined the correlation between men’s faithfulness judgments and the self-reported EPC behavior of the models. There was a significant difference between faithfulness judgments made from face and full person images (*t*_33_ = 3.28 *p* = .002, face: *X*±SD = 4.46±0.64, full: *X*±SD = 4.75±0.46), so we examined correlations between faithfulness ratings and the models’ EPC behavior separately for each image type. There were no significant correlations between men's ratings of faithfulness and the self-reported EPC frequency of the female models being rated, for either face images (*r*_34_ = -0.08, *p* = .667) or full person images (*r*_34_ = -0.07, *p* = .707). Thus we replicated Rhodes et al.’s [11] finding that men cannot accurately judge quantitative variation in EPC behavior from face images in a new sample of participants, and found that providing full person images did not improve accuracy. Furthermore, we found no significant correlations between ratings of attractiveness and EPC behavior (face: *r*_34_ = 0.11, *p* = .537, full: *r*_34_ = -0.01, *p* = .979), or ratings of perceived trustworthiness and EPC behavior (face: *r*_34_ = 0.05, *p* = .784, full: *r*_34_ = -0.19, *p* = .281).

#### Attractiveness as a cue to faithfulness

To explore whether differences in the attractiveness of female face pairs were related to accuracy of men’s faithfulness judgments of those pairs, attractiveness scores for each model were averaged across participants and the difference in attractiveness (‘attractiveness difference’) between the models in each pair was calculated. We then correlated the attractiveness difference scores with the proportion of participants who correctly chose the most faithful model from that pair. We found no significant correlations between the attractiveness difference and the proportion of participants who correctly chose the most faithful model (*r*_17_ = -.15, *p* = .576). Nor were differences in rated femininity, averageness, or symmetry of the faces (available from Rhodes at el., [25]) significantly correlated with the proportion of participants who correctly chose the faithful model (femininity: *r*_34_ = -0.16, *p* = .534, averageness: *r*_34_ = 0.22, *p* = .397, symmetry: *r*_34_ = -0.02, *p* = .934). Therefore, although ratings of perceived faithfulness correlated positively with ratings of attractiveness (Table 1), neither attractiveness nor its components appear to have been used as cues to make faithfulness judgments in the forced choice task.

#### Perceived trustworthiness and faithfulness

There was a strong and significant correlation between ratings of trustworthiness and ratings of faithfulness (Table 1). We therefore investigated whether differences in perceived trustworthiness of the models’ faces were related to the proportion of participants who were able to accurately choose the more faithful model from each pair. We calculated the difference in perceived trustworthiness (hereafter ‘trustworthiness difference’) as described above for attractiveness difference. There was a strong and significant correlation between the trustworthiness difference and the proportion of participants who correctly chose the faithful model (*r*_17_ = .76, *p*< .001). Therefore, men may have used perceived trustworthiness as a cue to faithfulness.

## Experiment 2

Here we aimed to replicate the findings of Experiment 1 by having a new sample of male participants complete the faithfulness forced choice task using face images from Experiment 1. We also investigated whether individual variation in preference for faithfulness in a long-term partner is related to men’s accuracy in their faithfulness judgments. To test this, we had participants rate their preference for faithfulness in a potential long-term partner prior to completing a forced choice faithfulness task and compared these ratings with the accuracy of their faithfulness judgments. This experiment was initially part of a larger experiment designed to determine whether priming to an environment depicting sexual competition could improve accuracy. However, we have chosen not to present the priming results here due to concerns about the effectiveness of the priming manipulation. Details of this experiment can be found in (S2 File).

### Materials and methods

#### Participants

Sixty self-reported heterosexual, male participants of Western European descent, aged between 18 and 35 years of age were recruited from the University of Western Australia community and were awarded either psychology course credits or were remunerated with AU$5 for their participation. Participants were first provided with an information sheet detailing their role in the study and signed a consent form before participating.

#### General procedure

All testing took place on a MacBook Pro, 15 inch, 1440 x 900 pixel resolution screen. All experimental tasks were programmed and performed using SuperLab 4. All participants were tested in a private room with no experimenter present.

Participants began by completing a ‘Mate Preference Questionnaire’ that measured the importance of 10 mate choice related traits in a sexual partner including faithfulness [5]. These traits were rated on a 9-point Likert scale from 1 (‘Not at all important’) to 9 (‘Extremely important’). Once the questionnaire had been completed, participants completed the faithfulness forced choice task using faces, as described in Experiment 1. The images used in this experiment were the same as those used in Experiment 1.

### Results and discussion

Where variables did not meet the assumption of normality according to a Kolmogorov Smirnov test, parametric analysis was still used because *z* scores calculated from skewness and kurtosis values were less than 1.96 [39] and/or non-standardized residuals after parametric analysis were normally distributed. Non-parametric analyses did not alter our results and are presented in (S3 File) to allow comparison.

#### Accuracy of faithfulness judgments

**Fig 1:**
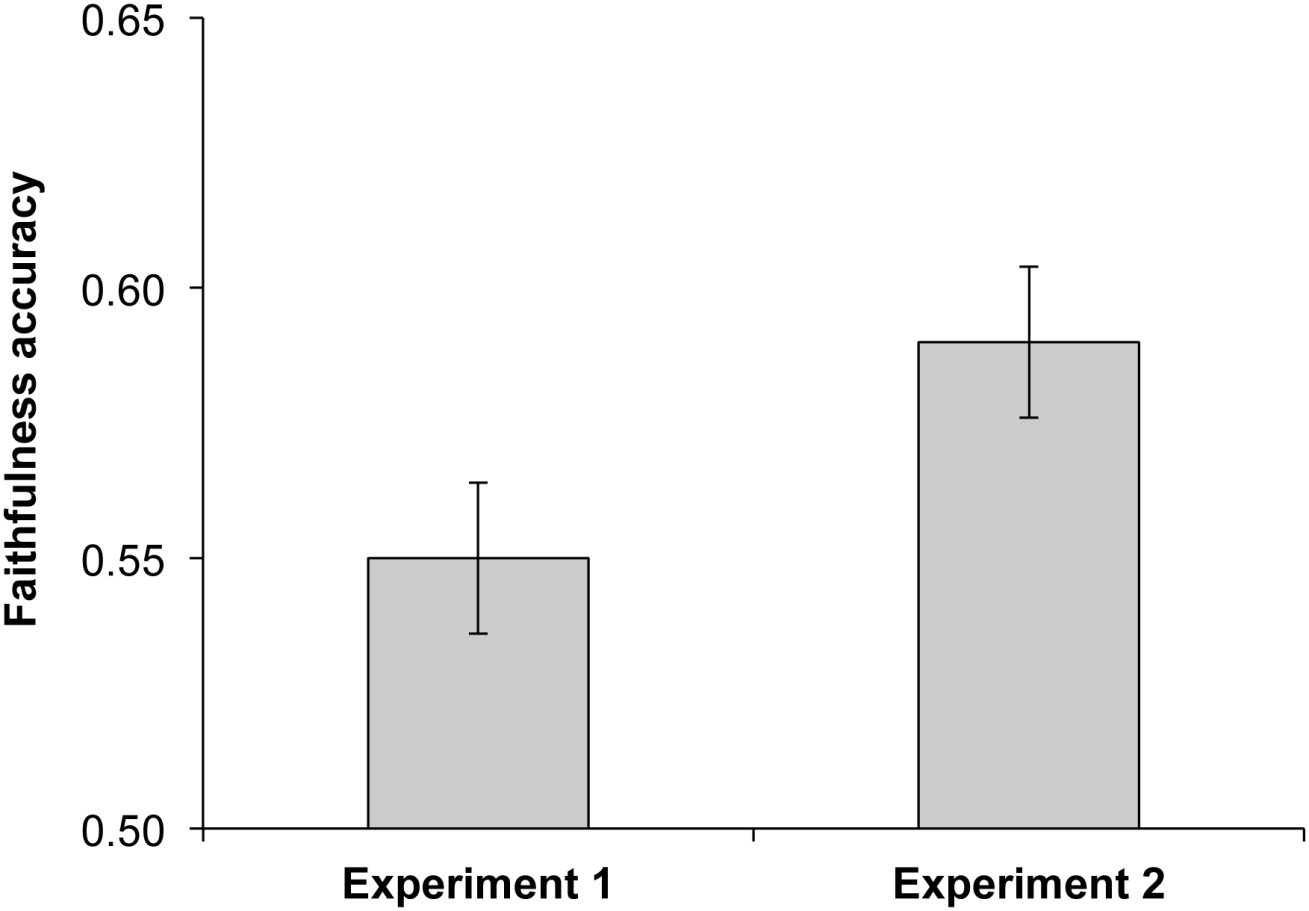
Faithfulness accuracy (proportion of trials in which the faithful model was correctly chosen in a forced choice task) in Experiment 1 and Experiment 2. The x-axis represents chance level (0.5) and S.E bars are shown.

The proportion of correct choices was defined as the proportion of trials in which the participant correctly chose the faithful model. An independent samples *t* test showed that performance was significantly above chance (0.5) (one sample t test: *t*_*59*_ = 6.71, *p*< .001, *X*±SD = 0.59±0.11) and the effect size was large (*r* = 0.66, 95% CI = 0.49–0.78). These results replicate the accuracy demonstrated in Experiment 1 with a new sample of participants (Fig 1).

As in Experiment 1, we found considerable variation between the 17 pairs of women in how likely participants were to choose the most faithful model of the pair (proportion of correct responses ranging from 0.20–0.83, *X*±SD = 0.59±0.19). However, despite the average proportion of correct responses falling below chance level (0.5) for only four of the 17 pairs, the proportion of correct responses across pairs was not significantly different from chance (one sample t test: *t*_16_ = 2.02, *p* = .060, effect size: *r* = 0.45, 95% CI = -0.06–0.77), thus replicating the findings of Experiment 1 that accuracy does not generalize across all face pairs.

Participant’s self-reported preferences for faithfulness were positively skewed (*X*±SD = 8.18±1.00) and were unrelated to accuracy of faithfulness judgments (*r*_60_ = .02, *p* = .884). Accuracy was also unrelated to preference for the other nine traits measured in the Mate Preference Questionnaire (desire for children, physical attractiveness, health, strength of family bonds, devotion, social status, ambition, parenting qualities and financial resources), all *r*s < .19, *p*s > .14.

#### Perceived trustworthiness and faithfulness

Using the trustworthiness difference scores from Experiment 1, we investigated whether the difference in perceived trustworthiness was related to the proportion of participants who accurately chose the more faithful model from each pair. The correlation was large and significant (*r*_17_ = 0.72, *p* = .001), again replicating the results of Experiment 1 with a new sample of participants.

## General Discussion

People show high consensus on many trait judgments, often from limited visual information, and these initial impressions can sometimes contain a kernel of truth [10,11,40]. Previous research suggests that men’s judgments of female faithfulness made from images of women show high consensus [12–14] but no accuracy [13]. Our results provide the first evidence that such judgments can contain a kernel of truth. When asked to choose the more faithful women from pairs of images, men chose the woman who had not reported any EPCs significantly above chance level. Accuracy of judgments from faces had a moderate-large effect size and was repeatable across two experiments using different samples of men. Accuracy of judgments from full person images also had a moderate effect size, but was not significant with our relatively small sample of model pairs.

Although men’s judgments contained a kernel of truth when they selected the more faithful of two women in a forced choice task, they showed no accuracy in rating individual women. Their judgments of faithfulness did not correlate with the self-reported EPC behavior of the models. This result replicates Rhodes et al’s [13], using a subset of the faces from that study but different participants. Furthermore, our results also suggest that the ability to accurately judge faithfulness at above-chance levels in a forced choice task does not generalize to all faces, being easier for some pairs of women than others. However, it is worth noting that the power of this analysis is low due to the limited number of 17 pairs. This resulted in wide confidence intervals on the effect size. Future research would do well to use more pairs of women to truly determine men’s accuracy in judgments of faithfulness. Similarly, using images of different women to those used in both of these experiments would provide a stronger test of the repeatability of men’s accuracy in faithfulness judgments.

We examined two main ways in which men’s accuracy in faithfulness judgments might be improved: through the use of a forced choice task and by using full person images. In Experiment 1, we found that men can show some accuracy in their judgments of faithfulness when completing a forced choice task. As our findings repeated those of Rhodes et al [13] in that our participants showed no accuracy in their judgments of female faithfulness in a ratings task, our study suggests that the use of forced choice tasks may be useful in revealing discrimination accuracy where ratings tasks fail to do so. In a ratings task, two similar items could be given the same rating, but when forced to choose one over the other, people might exhibit a consistent (although subtle) preference.

We found no evidence that accuracy was better for full person than face images. If anything, the evidence for accuracy was clearer for faces, because accuracy was significant for faces, but only approached significance for full person images. This result seems somewhat paradoxical given that the full person images contained faces. However, the faces in these images were considerably smaller than in the face images themselves, potentially making it more difficult to use any honest facial cues to faithfulness. The visual complexity of full person images would also have reduced the time available to process information from the faces.

In Experiment 2, we replicated the findings of Experiment 1 and found that men’s accuracy was significantly above chance level, suggesting that their accuracy in faithfulness judgments when completing a forced choice task is robust. The results of Experiment 2 also rule out the possibility that the accuracy seen in Experiment 1 (in the forced choice task) actually results from having seen all the faces before (in the ratings task completed prior to the forced choice task). However, as in Experiment 1, we found that the ability to accurately judge faithfulness at above-chance levels in a forced choice task was easier for some pairs of women than others, and did not generalize to all face pairs. In addition, we found no evidence that men who show a greater preference for faithfulness in a potential partner show better accuracy then men who have less of a preference for faithfulness. However, our participants showed little variation in their preference for faithfulness with most participants rating faithfulness as a highly important trait in a potential partner, which could account for this result.

Previous research has indicated that attractiveness can act as an honest cue to women’s faithfulness [12,14]. However, we found no relationship between the model’s rated attractiveness and men’s accuracy in their judgments of faithfulness. Nor did participants use individual components of attractiveness to make their judgments of faithfulness. Whereas Rhodes et al. [11] found that women’s accurate judgments of male faithfulness were cued by a specific component of attractiveness, namely masculinity, we found no link between differences in ratings of femininity of the models and accuracy of faithfulness judgments. Nor did differences between models in other components of attractiveness (i.e. symmetry and averageness) correlate with accuracy. These results suggest that men do not use attractiveness as an cue to female faithfulness. We note, however, that the different judgments were made by different raters (to avoid carry-over effects), so this conclusion depends on good consensus of these judgments across raters.

Another trait that might relate to judgments of faithfulness is perceived trustworthiness. We found that the perceived trustworthiness of the models was related to men’s accurate judgments of faithfulness: If the faithful model was perceived as more trustworthy than the unfaithful model, participants were more likely to correctly choose her as the faithful model. This finding is not in line with evidence from Rhodes et al. [11] that indicated that women’s accurate judgments of male faithfulness were independent of their judgments of trustworthiness. In future research, one might provide a specific ‘trust scenario’ outside of a mate choice context (e.g. “How much would you trust this person with a secret?”) to further investigate the relationship between perceived trustworthiness, perceived faithfulness and men’s accuracy in faithfulness judgments, including the mechanisms by which these judgments are made.

Why perceptions of trustworthiness are related to faithfulness judgments for men but not for women is unknown. One possibility is that, by forcing participants to discriminate between two faces, forced choice tasks may artificially induce preferences where none would be detected using other methods, such as a ratings task. [41]. However, evidence suggests that trait preferences revealed in a forced choice task are not artificially different from those made from a rating task and these preferences predict ratings of both actual and ideal partner traits, whereas ratings tasks do not [42]. The use of a forced choice task may not force participants to discriminate between individuals based on traits that would other be overlooked, but instead could simply encourage them to make finer discriminations on the same traits as used to make ratings.

An alternative explanation for our finding is that judgments of faithfulness may be made using visual cues that are also used to make judgments of trustworthiness. For example, emotion expression has been found to influence a number of trait judgments including perceived trustworthiness [43,44]. Whether emotion expression might influence faithfulness judgments has not been explored. Although the models in this study posed with neutral facial expressions, neutral expressions can naturally resemble emotion expressions, which may in turn influence trait judgments (emotion overgeneralization [44,45,46–48]). Although trait judgments in our set of stimuli are unlikely to be strongly influenced by emotion overgeneralization (the images of our models did not capture behaviors or expressions that traditional indicate flirtatious or sexual behavior e.g. tilted head, eyebrow flashes or lip moistening [49]), it is possible that subtle differences in perceived affect of the models remain, even in passport style photographs. Certainly, more research is needed to determine the visual cues that men use to make their judgments of faithfulness, including emotion expression and other face gesture cues.

We have suggested that accuracy in faithfulness judgments could aid in assessing potential long-term partners to avoid cuckoldry. However, it could also play a role in assessing women’s willingness to engage in EPCs outside of their relationship. Mate poaching (whereby an individual attempts to engage another individual, already in a committed relationship, into a relationship or brief sexual encounter) is a prevalent mating tactic. Indeed, 60% of men admit to attempting to poach woman for a brief sexual encounter whilst 31% of women admit to being successfully poached from their committed relationship for a brief sexual encounter [26].

In summary, we show for the first time that men’s judgments of faithfulness from images of women can contain a kernel of truth when they are able to directly compare images in a forced choice task, although accuracy did not generalize to all pairs of women. Previously, accuracy in faithfulness judgments has only been found for women judging men’s faces [11]. It is striking that men were able to show any accuracy from images alone after only a brief presentation, considering that accuracy in faithfulness judgments made from behavioral information is relatively poor [7,13].

## Supporting Information

S1 File Non-parametric analysis of Experiment 1. (DOCX)

S2 File Priming experiment. (DOCX)

S3 File Non-parametric analysis of Experiment 2. (DOCX)
