# Supplementary material for: Men’s Sexual Faithfulness Judgments May Contain a Kernel of Truth
Source: PLoS One. 2015 Aug 5;10(8):e0134007. doi: 10.1371/journal.pone.0134007 (PMC4526544; doi:10.1371/journal.pone.0134007)
Supplement: S1 File — (DOCX) [file pone.0134007.s001.docx]

*Accuracy of faithfulness judgements*

The proportion of correct choices was defined as the proportion of times the participant correctly chose the faithful model. There was no significant difference in accuracy when judgements were made from face and full person images (Mann-Whitney U Test, *U*_43_= -0.62, *p*= .536). Overall, accuracy was significantly above chance (0.5) (Wilcoxon Signed Rank test: *W*_43_= 2.75, *p*= .006, *X*±SD= 0.55±0.11). Although there was no significant difference in performance on faces and full person images, planned tests showed that performance was significantly above chance for judgements from face images (Wilcoxon Signed Rank Test: *W*_21_= 2.18, *p*= .030, *X*±SD= 0.57±0.12) but only marginally significant from full person images (Wilcoxon Signed Rank Test: *W*_22_= 1.71, *p*= .087, *X*±SD= 0.54±0.11). These results show that men’s judgements of faithfulness have a kernel of truth, at least for judgments from women’s faces.

The proportion of times that participants correctly chose the most faithful model from each of the 17 pairs was calculated and we found considerable variation between the 17 pairs of women in how likely participants were to choose the most faithful model of the pair (proportion of correct responses ranging from 0.29 - 0.86, *X*±SD= 0.57±0.18). Although the average proportion of correct responses fell below chance level (0.5) for only six of the 17 pairs, further analysis found that the proportion of correct responses across pairs was not significantly different from chance (Wilcox Signed Rank Test: *W*_17_= 1.45, *p*= .148).

To examine men’s accuracy in faithfulness judgements in the ratings task, we examined the correlation between men’s faithfulness judgements and the self-reported EPC behaviour of the models. There was a significant difference between faithfulness judgements made from face and full person images (Wilcoxon Signed Rank Test: face: *W*_34_= -3.08, *p*= .002, *X*±SD= 4.46±0.64, full: *X*±SD= 4.75±0.46), so we examined the correlation between faithfulness ratings and the models’ EPC behaviour separately for each image type. There were no significant correlations between men's ratings of faithfulness and the self-reported EPC frequency of the female models being rated, for either face images (Spearman’s *r*_34_= -0.26, *p*= .135) or full person images (Spearman’s *r*_34_= -0.14, *p*= .419).

*Attractiveness as an honest cue of faithfulness*

We investigated whether attractiveness ratings of the models were related to the proportion of participants who accurately chose the faithful model from the pair. There were no significant correlations between the attractiveness difference and accuracy (face: Spearman’s *r*_17_= -0.16, *p*= .542, full-person: Spearman’s *r*_17_= -0.12, *p*= .656). Nor did differences in components of attractiveness (facial femininity/averageness/symmetry) correlate significantly with accuracy (femininity: Spearman’s *r*_34_= -0.20, *p*= .453, averageness: Spearman’s *r*_34_= 0.22, *p*= .387, symmetry: Spearman’s *r*_34_= 0.04, *p*= .873).

*Trustworthiness as an honest cue of faithfulness*

There were significant correlations between the trustworthiness difference of the models and the proportion of participants who accurately chose the most faithful model (face: Spearman’s *r*_17_= 0.76, *p*< .001, full-person: Spearman’s *r*_17_= 0.83, *p*< .001): If the faithful model looked more trustworthy than the unfaithful model, participants were more likely to correctly choose the faithful model as the more faithful of the pair.
