# Supplementary material for: Men’s Sexual Faithfulness Judgments May Contain a Kernel of Truth
Source: PLoS One. 2015 Aug 5;10(8):e0134007. doi: 10.1371/journal.pone.0134007 (PMC4526544; doi:10.1371/journal.pone.0134007)
Supplement: S2 File — (DOCX) [file pone.0134007.s002.docx]

*Accuracy of faithfulness judgements*

The proportion of correct choices was defined as the proportion of trials on which the participant correctly chose the faithful model. We found that accuracy was significantly above chance (0.5) (Wilcoxon Signed Rank Test, *W_60_*= 5.31, *p*< .001, *X*±SD= 0.59±0.11).

As in Experiment 1, we found considerable variation between the 17 pairs of women in how likely participants were to choose the most faithful model of the pair (proportion of correct responses ranging from 0.20 - 0.83, *X*±SD= 0.59±0.19). However, despite the average proportion of correct responses falling below chance level (0.5) for only four of the 17 pairs, the proportion of correct responses across pairs was not significantly different from chance (Wilcox Signed Rank Test: *W_17_*= 1.63, *p*= .103).

Participant’s self-reported preferences for faithfulness were highly positively skewed (*X*±SD= 8.18±1.00) and were unrelated to accuracy of faithfulness judgments (Spearman’s *r*_60_= .01, *p*= .997). Accuracy was also unrelated to preference for the other nine traits measured in the Mate Preference Questionnaire, all Spearman’s *r*s < .22, *p*s > .09.

*Perceived trustworthiness as an honest cue of faithfulness*

Using the trustworthiness difference scores from Experiment 1, we investigated whether the difference in perceived trustworthiness was related to the proportion of participants who accurately chose the more faithful model from each pair. The correlation was large and significant (Spearman’s *r*_17_= 0.67, *p*= .003), again replicating the results of Experiment 1 with a new sample of participants.
