# Supplementary material for: Men’s Sexual Faithfulness Judgments May Contain a Kernel of Truth
Source: PLoS One. 2015 Aug 5;10(8):e0134007. doi: 10.1371/journal.pone.0134007 (PMC4526544; doi:10.1371/journal.pone.0134007)
Supplement: S3 File — (DOCX) [file pone.0134007.s003.docx]

The aim of this experiment was to assess whether the accuracy of men’s judgments of faithfulness from faces could be improved by priming them to an environment depicting sexual competition. Men completed the faithfulness forced choice task from Experiment 1 both before and after exposure to one of three image conditions. These images depicted either a mating environment in which there was sexual competition between males (sexual competition condition), a mating environment in which there was no sexual competition between males (no sexual competition condition), or control images (control condition). We predicted that men’s accuracy in faithfulness judgments would increase after exposure to images depicting sexual competition. We further examined the relationship between perceived trustworthiness and accuracy in faithfulness judgments.

We also investigated whether individual variation in preference for faithfulness in a long-term partner is related to men’s accuracy in their faithfulness judgments. Participants rated their preference for faithfulness in a potential long-term partner prior to completing a forced choice faithfulness task and these ratings were compared with the accuracy of their faithfulness judgments.

We chose not to report these findings in the main paper due to methodological limitations. Although the images used to prime participants to a sexual environment have been successfully used previously in human sperm competition research [1], we did not assess in this experiment whether our priming manipulation was successful. Furthermore, we had participants complete the forced choice task both prior to and after exposure to priming images, which may have led to carryover effects in the post exposure forced choice task. As such, we present the results of the pre-exposure forced choice task only in the main paper in order to replicate the results of Experiment 1. The methodology and results of our original experiment are presented below.

**Materials and methods**

**Participants**

Sixty self-reported heterosexual, male participants of Western European descent, aged between 18 and 35 years of age were recruited from the University of Western Australia community and were awarded either psychology course credits or were remunerated with AU$5 for their participation. Participants were first provided with an information sheet detailing their role in the study and signed a consent form before participating. The information sheet stated clearly that participants might be exposed to sexually explicit images and that any participant uncomfortable with viewing such images should not continue with the experiment.

**General procedure**

Participants began by completing a ‘Mate Preference Questionnaire’ that measured the importance of 10 mate choice related traits in a sexual partner including faithfulness [2]. These traits were rated on a 9-point Likert scale from 1 (‘Not at all important’) to 9 (‘Extremely important’). Once the questionnaire had been completed, participants completed the faithfulness forced choice task using faces, as described in Experiment 1. Participants were then exposed to one of three sets of images: Twenty participants were allocated to a sexual competition image condition, 20 to a no sexual competition image condition and 20 to a control image condition. Four explicit multi-male with female images (two men, one woman) were used for the sexual competition condition and four female only explicit images (three women) used for the no sexual competition condition. Participants assigned to the control condition viewed four pictures of pedigree dogs found on the Internet. An information screen instructed participants to initiate a slideshow of their assigned images by pressing the space bar and to look carefully at the images. Following the protocol of Little, DeBruine and Jones [3], each of the assigned four images was shown for 3 s and was repeated two times (for a total of eight images and an total exposure time of 24 s). Once all images had been viewed, participants then repeated the faithfulness forced choice task. Finally, they completed another ‘Mate Preference Questionnaire’ to check for reliability in self-reported preference for faithfulness. This questionnaire was identical to the one completed at the start of the study except for the order in which the questions were presented.

**Results and discussion**

**Accuracy of faithfulness judgments**

We used a two-way repeated measures ANOVA, with test time (pre and post-exposure to images) as a within participants variable, image condition (sexual competition, no sexual competition and control) as a between participants variable, and faithfulness accuracy (proportion of trials in which the more faithful model was chosen) as the dependent variable. There was no significant main effect of test time (*F*_1,57_= 0.07, *p*= .792, pre: *X*±SD= 0.59±0.11, post: *X*±SD= 0.60±0.12) and no main effect of image condition (*F*_2,57_= 2.13, *p*= .128, sexual competition: *X*±SD= 0.57±0.10, no sexual competition: *X*±SD= 0.62±0.07, control: *X*±SD= 0.60±0.10). Nor was there any significant interaction between test time and image condition (*F*_2,57_= 0.93, *p*= .402) (Fig. 1). These results provide no evidence that accuracy of faithfulness judgments increase in accuracy when men are primed to consider sexual competition. Overall, accuracy was significantly above chance level (*t*_59_= 8.33, *p*< .001, *X*±SD= 0.60±0.09). The effect size was large (*r*= 0.74, 95% CI= 0.59-0.83). These results replicate the accuracy demonstrated in Experiment 1 with a new sample of participants.

Participant’s self-reported preference for faithfulness showed good test-retest reliability (*r*_60_= .76, *p*< .001). However, this preference was unrelated to accuracy of faithfulness judgments (*r*_60_= .05, *p*= .708). Accuracy was also unrelated to preference for the other nine traits measured in the Mate Preference Questionnaire, all *r*s < .11, *p*s > .40.

**Perceived trustworthiness and faithfulness**

As there was no significant difference in the proportion of times that participants chose the faithful model between the pre and post exposure tasks (see ANOVA above), we calculated the overall mean for the proportion of times that participants correctly chose the faithful model and correlated this variable with the trustworthiness difference score for each pair which was calculated in Experiment 1. The correlation was large and significant (*r*_17_= 0.73, *p*= .001).

1. Kilgallon SJ and Simmons LW (2005) Image content influences men's semen quality. Biology Letters 1: 253-255. doi:10.1098/rsbl.2005.0324.

2. Buston PM and Emlen ST (2003) Cognitive processes underlying human mate choice: The relationship between self-perception and mate preference in Western society. Proceedings of the National Academy of Sciences 100: 8805-8810. doi:10.1073/pnas.1533220100.

3. Little AC, DeBruine LM and Jones BC (2013) Environment contingent preferences: Exposure to visual cues of direct male-male competition and wealth increase women's preferences for masculinity in male faces. Evolution and Human Behavior 34: 193-200. doi:10.1016/j.evolhumbehav.2012.11.008.
